# Assessing healthcare cost changes associated with transitioning away from cigarette smoking using healthcare claims data: an exploratory study among adult male patients with COPD

**DOI:** 10.1186/s12954-024-01141-4

**Published:** 2024-12-23

**Authors:** Mingda Zhang, Hui G. Cheng, Brendan Noggle, Jud C. Janak, Megan Richards, David Smith

**Affiliations:** 1 Altria Client Services LLC, 601 E. Jackson Street, Richmond, VA 23219 USA; 2 Merative LP, 75 Binney St. 4th Floor, Cambridge, MA 02142 USA

**Keywords:** Real-world evidence, Smoking cessation, Smokeless tobacco, Tobacco harm reduction, Chronic obstructive pulmonary disease, Healthcare claims, Healthcare cost, Health economics

## Abstract

**Background:**

The assessment of potential health effects of switching from cigarette smoking to non-combustible tobacco products has important implications for public health and regulatory decisions. Robust epidemiological evidence requires long-term follow-up of a large number of individuals. Real-world evidence derived from health records has the potential to help fill the gap in the interim. To our knowledge, this is the first study using individual-level healthcare claims data to assess the potential impact of transitioning from cigarette smoking to smokeless tobacco on short-term direct healthcare costs.

**Methods:**

We conducted a retrospective cohort study of adult male patients with COPD who smoked cigarettes at baseline using the MarketScan^®^ Databases. We compared changes in direct healthcare costs between the 12-month periods before (baseline) and after the index date (follow-up) across three cohorts: continued smoking (CS), quit all tobacco (QT), or switched to smokeless tobacco (SW), using a non-linear difference-in-differences model with average marginal effects.

**Results:**

A total of 23,427 COPD patients were included (CS: 11,167; QT: 12,013; SW: 247). At baseline, the QT cohort had the highest total average healthcare costs ($43,771), followed by SW ($38,419), and CS ($27,149). The unadjusted difference-in-differences model revealed no statistically significant differences in total healthcare cost changes when comparing the QT or SW cohorts to the CS cohort (-$1,532 [95% CI: -$3,671, $608] for the QT cohort, and -$452 [95% CI: -$15,415, $14,511] for the SW cohort). After adjusting for Deyo-Charlson Comorbidity Index and COPD exacerbation, assuming patients had two comorbidities and exacerbations, the QT cohort had greater reduction in total healthcare costs compared to the CS cohort (-$2,910 dollars [95% CI: -$4,485, $-1,335]). The same trend was observed for the SW cohort, although the estimate was not statistically significant (-$5,312 [95%CI: -$11,067, $442], *p* = 0.08).

**Conclusions:**

This study demonstrated the feasibility of using administrative claims to conduct real-world evidence studies on the harm-reduction potential of non-combustible tobacco products and found evidence suggesting reductions in direct healthcare costs after quitting tobacco or switching to smokeless tobacco among patients with COPD. Based on the learnings and limitations identified during the study, we propose concrete recommendations to improve future observational studies by integrating additional real-world healthcare data from multiple data sources.

**Supplementary Information:**

The online version contains supplementary material available at 10.1186/s12954-024-01141-4.

## Background

While cigarette smoking remains the leading cause of preventable morbidity and mortality in the United States (US) [1], concerns about the detrimental health effects have motivated many adults who smoke cigarettes to quit or switch to non-combustible products (NCP). Adult smoking prevalence has declined steadily from 52.0% and 34.1% in 1965 [1] for men and women, respectively, to 13.1% and 10.1% in 2021 [2]. The U.S. Food and Drug Administration (FDA) and other public health authorities agree that there is a broad “continuum of risk” among tobacco products, with combustible cigarettes at the highest end, NCP at the lower end, and complete cessation at the lowest end of that spectrum [3–5]. Consistent with FDA’s comprehensive plan announced in 2017 [6], for adults who smoke cigarettes who do not want to or are unable to quit, switching to NCP may offer a reduced-risk alternative to combustible tobacco products [4]. Indeed, there has been a growing trend of using and completely switching to NCP among adults who smoke cigarettes in recent years, coupled with an accelerated decline in cigarette smoking [7]. In 2023, over 40% of US adults who used tobacco have used NCP either exclusively or in combination with other products [7].

Epidemiological evidence remains the gold standard for assessing the public health impact of transitioning from cigarette smoking to NCP. The FDA has approved some traditional NCP like snus and moist smokeless tobacco as modified risk tobacco products (MRTP), relying heavily on epidemiological evidence [8]. Nonetheless, epidemiologic studies on the potential health effects of switching from smoking to modern NCP like electronic nicotine delivery systems (ENDS) and nicotine pouches face many challenges including the need for relatively long follow-up period for a large number of individuals. Existing studies are primarily based on self-reported data from national surveys such as the National Health Interview Survey (NHIS) [9] and the Population Assessment of Tobacco and Health (PATH) Study [10]. Real-world evidence derived from administrative health claims and electronic health records can help provide interim evidence in a more timely and cost-efficient way until sufficient epidemiological evidence becomes available. More specifically, in addition to being a meaningful measure, the difference in total healthcare costs can serve as an indicator for the impact on overall health status in the context of tobacco harm reduction.

Chronic obstructive pulmonary disease (COPD) is primarily caused by cigarette smoking [1]. Previous studies have shown that patients with COPD who switched to NCP like ENDS [11] and heated tobacco products [12] had better health outcomes and lower COPD exacerbations than those who continued smoking cigarettes. COPD exacerbations and COPD-related comorbidities are associated with a lower health related quality of life, increased healthcare costs, and increased risk of mortality [13]. We focused this study on smokeless tobacco (ST, including moist smokeless tobacco, dip, chewing tobacco, or snus) because claim codes did not exist for modern NCP like ENDS and nicotine pouches at the initiation of this study, and on male patients with COPD because ST is predominantly used by men in the US [2].

The objective of this study was to explore changes in direct healthcare costs among adult male patients with COPD who continue cigarette smoking (CS), who switched to smokeless tobacco products (SW), and who quit all tobacco (QT).

## Methods

### Study design and data sources

This was a retrospective cohort study using the Merative™ MarketScan^®^ Research Databases (MarketScan^®^ Databases) which include administrative claims data for patients insured commercially or through Medicare (see Supplemental File I for a more detailed description). International Classification of Diseases, Ninth/Tenth Edition, Clinical Modification (ICD-9/10-CM) codes were used to identify patients for each cohort.

### Study population and cohort identification

Patients in the MarketScan^®^ Databases who met all the following inclusion criteria detailed below were included in the study:

- Men with at least one ICD-9-CM or ICD-10-CM diagnosis or procedure code indicating current tobacco use or nicotine dependence between January 1, 2011, and December 31, 2022.
- Adults (≥ 18 years of age) on the earliest diagnosis or procedure claim for current tobacco use.
- No evidence of switching to ST or quitting (see Supplemental File II for ICD codes used to identify tobacco product use status) prior to January 1, 2015.
- Continuous enrollment with medical and pharmacy benefits in the 12 months prior to the index date (the baseline period) and 12 months following the index date (the follow-up period).
- At least one claim with an ICD-9-CM or ICD-10-CM diagnosis for COPD (ICD-9-CM: 491.xx, 496; ICD-10-CM: J41.x, J42, J44.1, J44.9) in the baseline period.

Eligible patients with COPD were stratified into three cohorts based on smoking behaviors:

- Men who continued to smoke cigarettes (CS cohort): Patients who continued to smoke cigarettes with no indication of smoking cessation or switching to ST.
- Men who smoked cigarettes and then quit tobacco products (QT Cohort): Patients who had evidence of cigarette smoking and subsequently quit smoking without indication of subsequent use of any tobacco products (both combustible and NCP).
- Men who smoked cigarettes and then switched to ST (SW Cohort): Patients who had evidence of cigarette smoking and subsequently switched to ST with no indication of current cigarette smoking or cessation after switch to ST.

### Study time periods

**Fig. 1:**
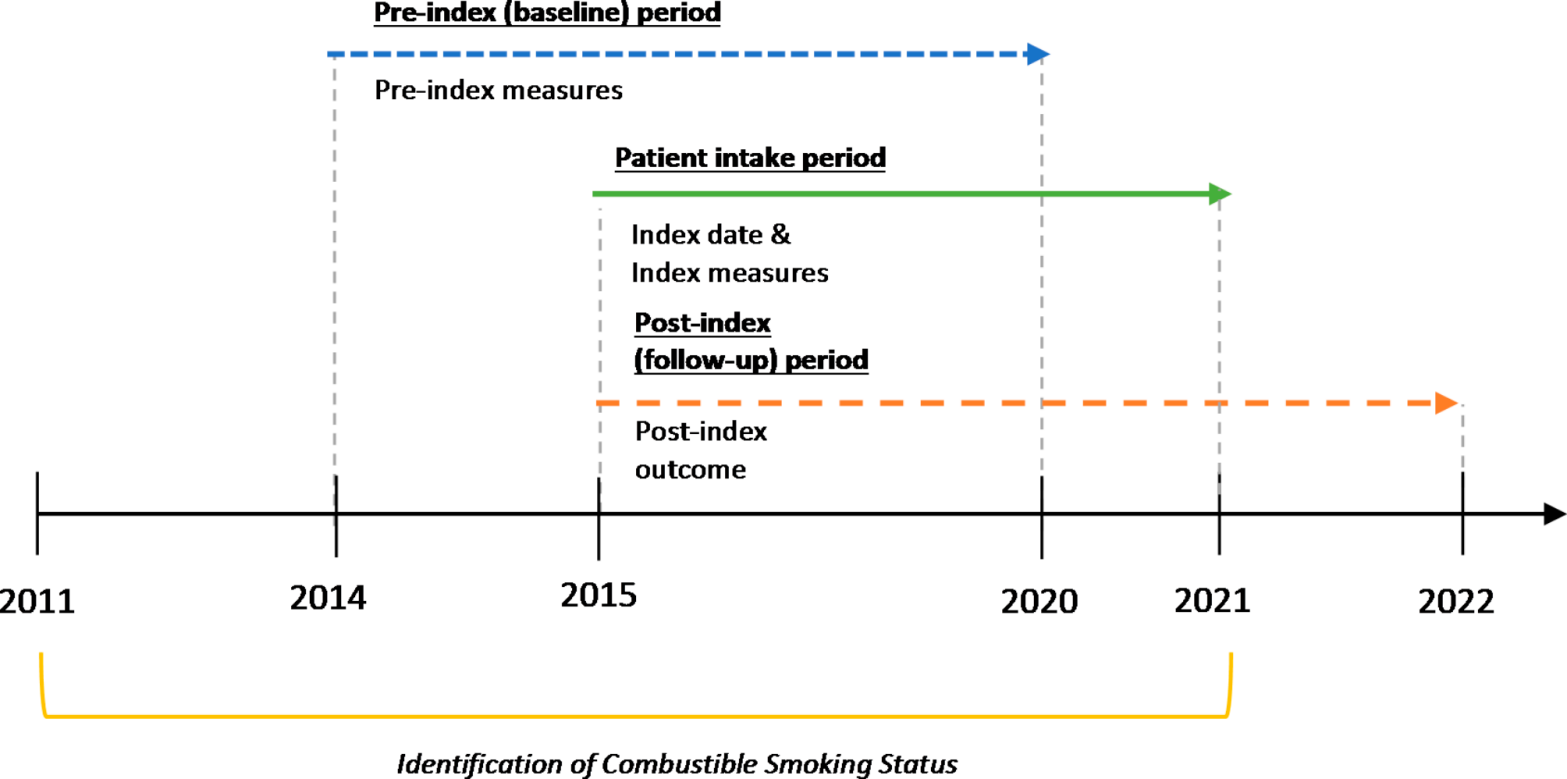
Study population identification and follow-up periods. Patient intake index dates occurred retrospectively over a 6-year period between 2015 to 2021, with a requisite 12-month baseline and 12-month follow-up period

To ensure all patients had a history of combustible smoking, patients for the study had at least one record indicating combustible smoking during the combustible smoking status assessment period between 2011 and 2021 (Fig. 1). The start of patient intake period was set to October 1, 2015, to align with the implementation of ICD-10-CM coding that includes new codes identifying ST use. The end of patient intake period was set to December 31, 2021, to maximize the number of patients while allowing for the 12-month follow-up period. The index date for the QT cohort was the date of the earliest record indicating smoking cessation. The index date for the SW cohort was the date of the earliest diagnosis or procedure code indicating a switch to ST. For men who continued to smoke, an index date was randomly assigned based on the distribution of index dates of the QT and SW cohorts.

### Outcome variables

Total healthcare costs were the outcomes of interest in this study. Total healthcare costs include all inpatient, outpatient, and pharmacy costs. Direct costs were based on paid amounts of adjudicated claims, including insurer and health plan payments as well as patient cost-sharing in the form of co-payment, deductible, and coinsurance. Cost for services provided under capitated arrangements were estimated using payment proxies based on paid claims at the procedure level using the MarketScan^®^ Databases. All dollar estimates were adjusted to 2020 dollars using the Medical Care Component of the Consumer Price Index. Direct costs were reported by type of service during the 12-month pre-index (baseline) and post-index (follow-up) periods. Direct cost results are presented separately for each study cohort.

### Baseline sociodemographic and clinical characteristics

Sociodemographic characteristics were measured on the index date and included age, U.S. Census Bureau geographic region, urban/rural residence, insurance plan type, primary payer, and the index year of the record. Baseline clinical characteristics were identified using ICD-10-CM codes for smoking-related comorbidities; results are presented for those conditions that had a prevalence ≥ 10% in at least one study cohort. COPD exacerbations were assessed both as a binary indicator of any COPD exacerbation during the baseline period and as the count of the total number of COPD exacerbations in both the baseline and follow-up periods [14]. Exacerbations recorded within a 14-day window were counted as a single episode. Deyo-Charlson comorbidity index (DCI) was calculated through an additive score based on the number and type of chronic conditions during the baseline period. A higher DCI score indicates increased likelihood of healthcare utilization and mortality [15]. All-cause healthcare utilization was described separately for each study cohort by type of service for both the baseline and follow-up periods in the Supplemental Files III to provide additional context.

### Statistical analysis

#### Descriptive statistics

Descriptive statistics were reported for baseline sociodemographic and clinical characteristics, unadjusted healthcare resource utilization, and unadjusted direct costs at baseline and follow-up for each cohort.

#### Non-linear difference-in-differences (DiD) regression

Combustible smoking is associated with many factors such as comorbid health outcomes and adverse health behaviors that may impact patients’ risk of COPD exacerbations and associated healthcare costs. Under the assumption that trends in pre-index costs are not meaningfully different across cohorts, non-linear difference-in-differences models can estimate the differences in changes in costs between the pre- and post-index periods associated with smoking behavior changes [16]. We chose the DiD model over conventional between-individual models because it is challenging to balance potential confounders when estimating the relationship between healthcare costs and changes in smoking behavior in an observational setting due to potential differential self-selection (e.g., those who have poorer health and higher healthcare expenditure choose to stop smoking). In the DiD model, each individual serves as his own control, therefore, all time-invariant variables are held constant [16].

The dependent variables for the regression model were total healthcare costs. Healthcare costs are known to be highly skewed and therefore were modeled using a generalized linear model with log link function and gamma error distribution [17]. For the small minority (< 5%) of patients with zero costs, their costs were imputed as one dollar. In the unadjusted model, we included smoking behavior cohort, time period, and the interaction of those two variables, depicted as follows:

$$\begin{aligned}&\text{l}\text{o}\text{g}\left({Cost}_{it}\right)\cr&\quad={\alpha\:}_{i}+\:{{\beta\:}_{1}(Smoking\:Behavior}_{i})\cr&\qquad +{{\beta\:}_{2}(Time}_{t})\cr&\qquad+{\beta\:}_{12}\left({Smoking\:Behavior}_{i}*{Time}_{t}\right)\cr&\qquad +{\epsilon\:}_{it}\end{aligned}$$

where $\:{\alpha\:}_{i}$ is the individual-specific intercept, $\:{\beta\:}_{1}$ is the difference between cohorts during the pre-index period, $\:{\beta\:}_{2}$ is the time trend in the control group (i.e., the CS cohort in this study), $\:{\beta\:}_{12}$ is the differences in changes across cohorts over time, and $\:{\epsilon\:}_{it}$ is the individual- and time-specific error term.

Separate models were run to compare the two different smoking behavior change cohorts (QT and SW) with the continuing smoking cohort (CS). Time period was defined as pre-index (reference) and post-index. The differences in changes in costs across cohorts (i.e., SW or QT as compared to CS) was estimated by the interaction term in the model. Given the complexity of interpreting an interaction term in non-linear models [16, 18], the approach recommended by Puhani [19] to report the average marginal effect of the interaction term was implemented. The Puhani average marginal effect estimate is the difference in the predicted direct costs in the post-index period associated with smoking behavior change (i.e., quitting or switching to ST) compared with the predicted direct cost if the patient continued to smoke cigarettes. A negative dollar value indicates an estimated reduction in direct healthcare costs for patients with COPD who either quit all tobacco products or switched to ST. Model coefficients with robust sandwich estimator standard errors were reported for each model as well as the average marginal effect and 95% confidence interval.

It was assumed that the difference between the average of the baseline costs and the average of the follow-up outcomes are the same for both cohorts with behavior changes (QT and SW cohorts) and control cohort (CS cohort).

#### Adjusted models and sensitivity analysis

Diagnosis of new or worsening of existing health conditions other than COPD exacerbation could have prompted some patients to change their smoking behavior [20, 21]. For example, it has been shown that adults with recent diagnoses of stroke, cancer, lung disease, heart disease, or diabetes mellitus were 3.2 times more likely to quit smoking than individuals without new diagnoses [21]. Previous research has also shown that adults who recently quit smoking are more likely to utilize more healthcare services within a year than those who smoke cigarettes [23]. Because of this phenomenon, studies among adults who recently quit smoking like ours may underestimate the beneficial effects of smoking cessation [24]. Since inflated healthcare costs immediately prior to and/or after the smoking behavior change for the QT and SW cohorts would violate the parallel trends assumption with the CS cohort, we re-ran the DiD models adding the total number of COPD exacerbations and DCI at any given time (pre-index or post-index) as covariates to account for the potential effect of COPD exacerbations and comorbidities (Adjusted Model). To estimate the average marginal effect estimate, we assumed a conservative two comorbidities and two exacerbations when comparing QT and SW cohorts with the CS cohort, respectively.

As a sensitivity analysis, we re-ran the DiD model excluding patients who had a COPD exacerbation in the 3 months prior to the index date, during which more patients who changed their smoking behavior likely were experiencing worsening COPD and disproportionate health care expenditures compared to patients who continued to smoke (Sensitivity Analysis).

## Results

**Fig. 2:**
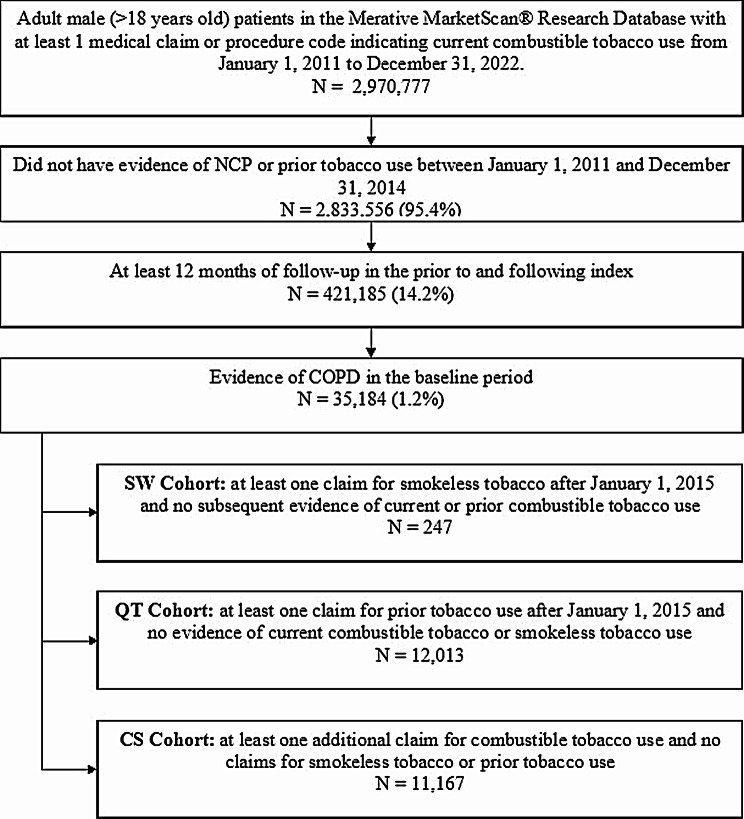
Flowchart of Patient Cohort Classification. Patients with COPD were screened into one of the three cohorts based on smoking history and tobacco use transitions during the intake period

Among 2,970,777 adult male (> 18 years old) patients with a smoking history in the study dataset, there were 11,167 patients in the CS cohort, 12,013 patients in the QT cohort, and 247 patients in the SW cohort eligible based on the inclusion and exclusion criteria (Fig. 2).

### Demographic and clinical characteristics

Patients in the QT cohort were slightly older than patients in the SW or CS cohorts (64.7, 62.4 and 62.1 years, respectively) (Table 1). The SW cohort was more likely to live in the South or in a rural area than the QT or CS cohorts. Compared to CS, patients in the SW cohort had a higher prevalence of asthma (20% vs. 10%), obesity (28% vs. 17%), osteoarthritis (30% vs. 21%), and sleep apnea (26% vs. 17%) prior to switching, whereas patients in the QT cohort had a higher prevalence of cancer (21% vs. 13%), emphysema (19% vs. 10%), and heart failure (17% vs. 10%) prior to quitting. The mean DCI score was slightly higher for patients in the QT cohort [3.1 (SD: 2.3)] compared than those in the SW [2.9 (SD: 2.3)] or CS cohorts [2.6 (SD: 2.2)]. These baseline differences are consistent with higher healthcare utilization and costs around the time of smoking cessation reported in the literature [25, 26].

**Table 1:** Baseline Sociodemographic and Clinical Characteristics

	SW Cohort	QT Cohort	CS Cohort
	*N* = 247	*N* = 12,013	*N* = 11,157
	Mean (SD)/N (%)	Mean (SD)/N (%)	Mean (SD)/N (%)
**Age (Mean**, **SD)**	62.4 (12.0)	64.7 (10.0)	62.1 (10.7)
**Geographic region (N**, **%)**			
Northeast	14 (5.7%)	1,895 (15.8%)	1,695 (15.2%)
North Central	91 (36.8%)	5,335 (44.4%)	4,533 (40.6%)
South	130 (52.6%)	4,008 (33.4%)	4,293 (38.4%)
West	12 (4.9%)	765 (6.4%)	638 (5.7%)
Unknown	(0.0%)	10 (0.1%)	8 (0.1%)
**Residence in an urban/rural area (N**, **%)**			
Urban	167 (67.6%)	9,702 (80.8%)	8,954 (80.2%)
Rural	79 (32.0%)	2,282 (19.0%)	2,192 (19.6%)
Unknown	1 (0.4%)	29 (0.2%)	21 (0.2%)
**Insurance plan type**^**1**^**(N**, **%)**			
Comprehensive	50 (20.2%)	3,331 (27.7%)	2,998 (26.9%)
EPO/PPO	137 (55.5%)	5,737 (47.8%)	5,443 (48.7%)
HMO	25 (10.1%)	1,222 (10.2%)	1,057 (9.5%)
POS/POS with capitation	9 (3.6%)	528 (4.4%)	562 (5.0%)
CDHP/HDHP	26 (10.5%)	1,108 (9.2%)	991 (8.9%)
Other/unknown	(0.0%)	87 (0.7%)	116 (1.0%)
**Payer (N**, **%)**			
Commercial	145 (58.7%)	6,223 (51.8%)	6,640 (59.5%)
Medicare supplemental	74 (30.0%)	4,331 (36.1%)	3,648 (32.7%)
Medicare advantage	28 (11.3%)	1,459 (12.2%)	879 (7.9%)
**Index year (N**, **%)**			
2015	24 (9.7%)	2,186 (18.2%)	2,781 (24.9%)
2016	64 (25.9%)	2,455 (20.4%)	2,597 (23.3%)
2017	52 (21.1%)	2,048 (17.1%)	1,910 (17.1%)
2018	28 (11.3%)	1,555 (12.9%)	1,381 (12.4%)
2019	28 (11.3%)	1,754 (14.6%)	1,133 (10.2%)
2020	36 (14.6%)	1,158 (9.6%)	812 (7.3%)
2021	15 (6.1%)	857 (7.1%)	553 (5.0%)
**Clinical Characteristics (N**, **%)**			
Any COPD exacerbations	122 (49.4%)	5,933 (49.4%)	4,878 (43.7%)
Count of COPD exacerbations	1.5 (1.0)	1.7 (1.1)	1.5 (0.9)
Acute respiratory illness (including pneumonia)	102 (41.3%)	5,104 (42.5%)	3,804 (34.1%)
Anxiety	60 (24.3%)	1,905 (15.9%)	1,837 (16.5%)
Asthma	49 (19.8%)	1,555 (12.9%)	1,078 (9.7%)
Atrial fibrillation	39 (15.8%)	1,768 (14.7%)	1,033 (9.3%)
Cancer	46 (18.6%)	2,491 (20.7%)	1,443 (12.9%)
Chronic Kidney Disease	23 (9.3%)	1,440 (12.0%)	981 (8.8%)
Coronary Heart Disease	80 (32.4%)	4,286 (35.7%)	3,097 (27.7%)
Depression	43 (17.4%)	1,662 (13.8%)	1,642 (14.7%)
Dyslipidemia	136 (55.1%)	7,398 (61.6%)	6,211 (55.6%)
Diabetes	72 (29.2%)	3,227 (26.9%)	2,880 (25.8%)
Emphysema	39 (15.8%)	2,227 (18.5%)	1,091 (9.8%)
Heart Failure	39 (15.8%)	2,022 (16.8%)	1,153 (10.3%)
Hypertension	181 (73.3%)	8,610 (71.7%)	7,509 (67.2%)
Nuclear cataract	17 (6.9%)	1,233 (10.3%)	969 (8.7%)
Obesity	69 (27.9%)	2,760 (23.0%)	1,930 (17.3%)
Osteoarthritis	75 (30.4%)	2,806 (23.4%)	2,409 (21.6%)
Peripheral arterial disease	23 (9.3%)	1,462 (12.2%)	1,171 (10.5%)
Sleep Apnea	65 (26.3%)	2,799 (23.3%)	1,921 (17.2%)
Sleep Disorder	25 (10.1%)	1,299 (10.8%)	1,038 (9.3%)
**Deyo-Charlson Comorbidity Index (Mean**, **SD)**	2.9 (2.3)	3.1 (2.5)	2.6 (2.2)

Abbreviations: EPO: Exclusive provider organization; HMO: Health maintenance organization; POS: Point of service; PPO: Preferred provider organization; CDHP: Consumer-driven health plan; HDHP: High deductible health plan; COPD, chronic obstructive pulmonary disease

### Healthcare costs

**Fig. 3:**
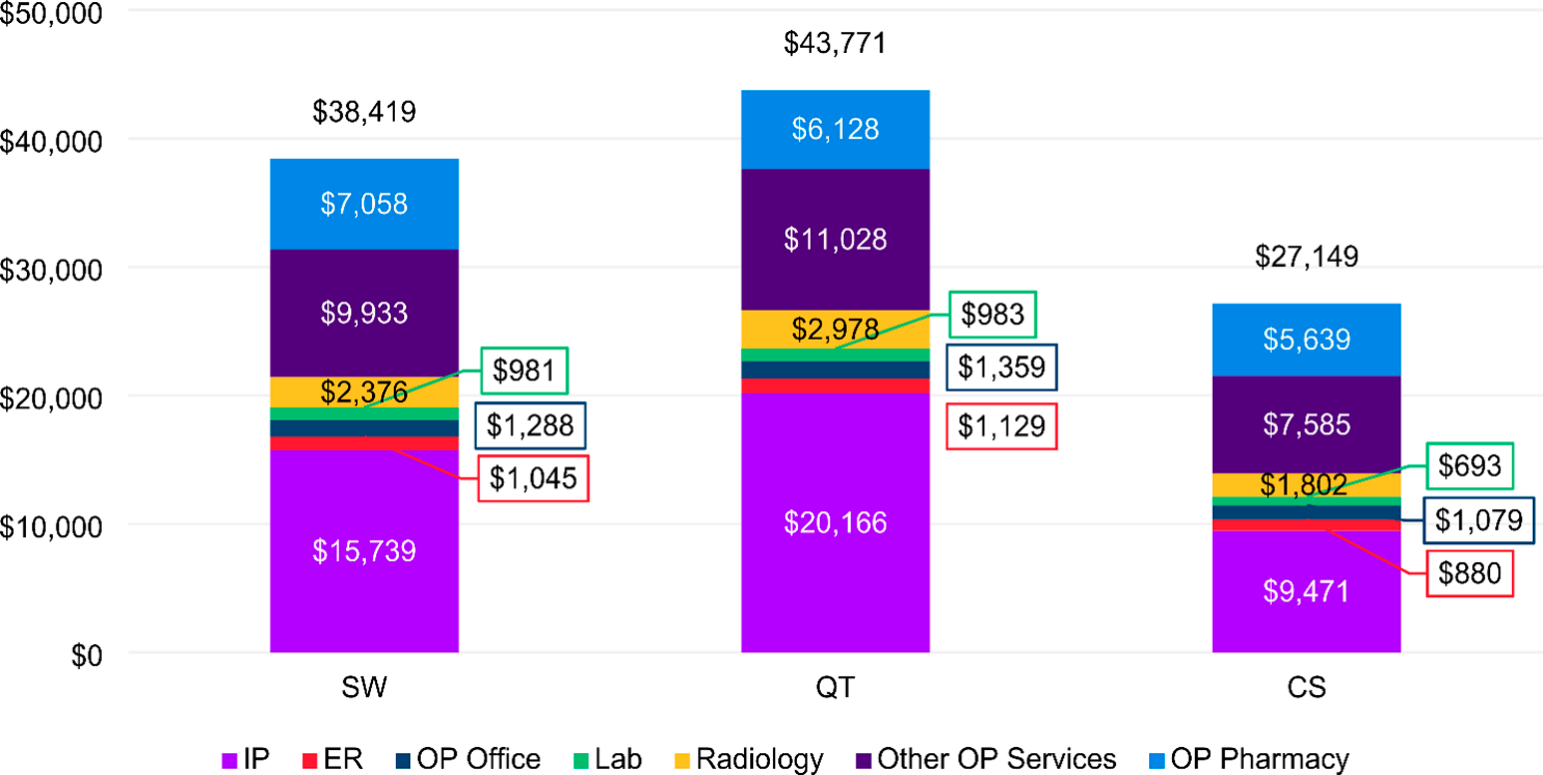
Average total healthcare costs in the baseline period, by cohort. Abbreviations: ER, emergency room; IP, inpatient; Lab, laboratory; OP, outpatient

During the baseline period, the average total healthcare costs were highest among patients in the QT cohort ($43,771), followed by the SW cohort ($38,419), and the CS cohort ($27,149) (Fig. 3). Inpatient admissions and other outpatient services accounted for the largest portions of healthcare costs for all three cohorts.

**Fig. 4:**
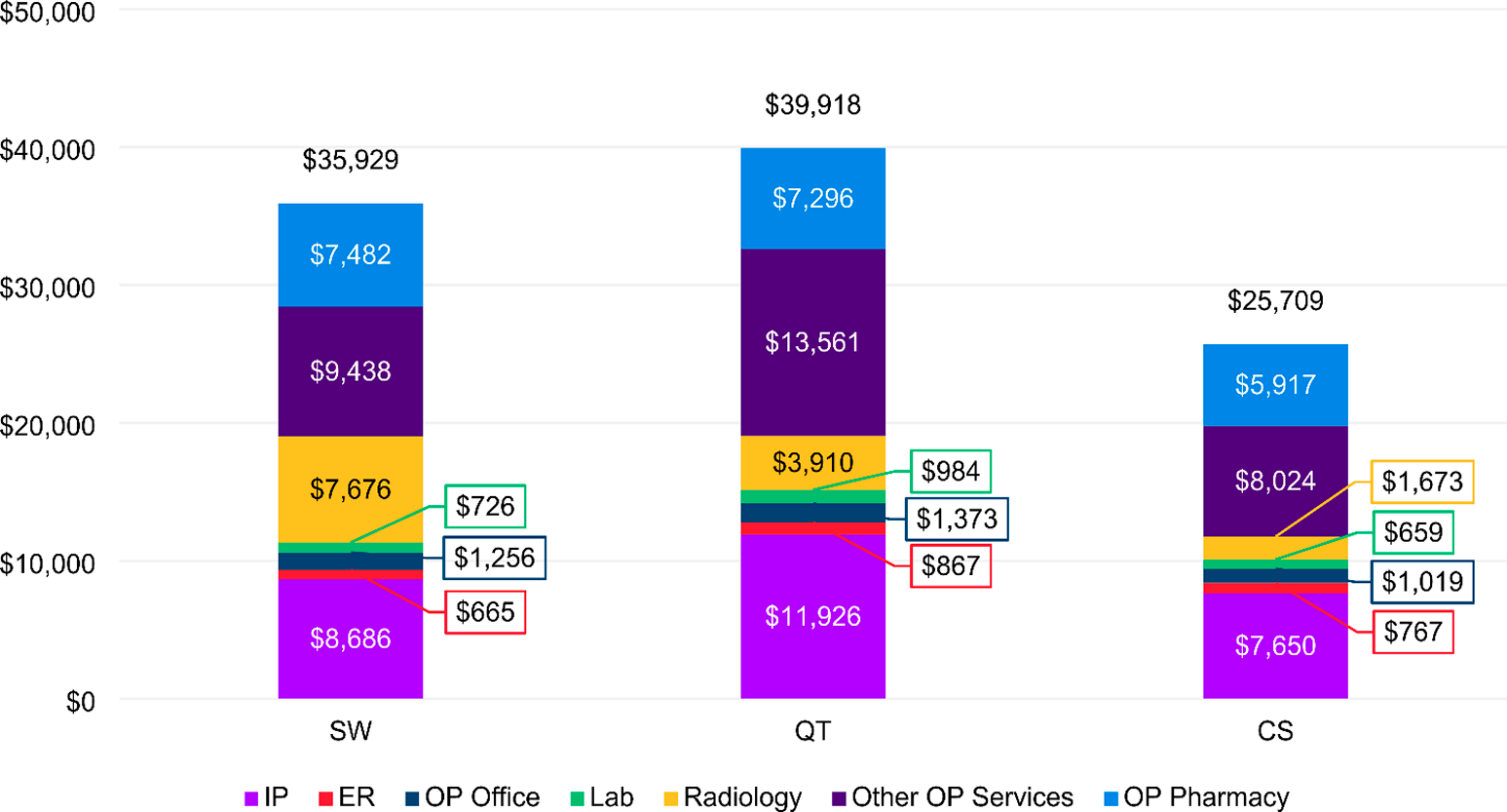
Average total healthcare costs in the follow-up period, by cohort. Abbreviations: ER, emergency room; IP, inpatient; Lab, laboratory; OP, outpatient

In the follow-up period, the average total healthcare costs declined for patients in all cohorts to $39,918, $35,929, and $25,709 for QT, SW and CS cohort, respectively (Fig. 4). The largest reduction was seen among the QT cohort ($3,854), followed by the SW cohort ($2,490), and the CS cohort ($1,440) (Figs. 3 and 4). Inpatient costs for patients in both the SW and QT cohorts were > 40% lower than baseline during follow-up, compared to a more moderate 19% reduction from baseline in the CS cohort. Interestingly, we observed a large increase in radiology costs among the SW cohort. In the follow-up period, patients in the SW cohort has on average $7,676 in radiology costs compared to $2,376 in the baseline, in contrast to the slight reduction in radiology utilization rate from 82.6 to 77.7% (Supplemental File III).

### DiD model results

Based on the unadjusted DiD regression results (Unadjusted Model), there were no statistically significant differences in total healthcare costs changes between the QT and CS cohort or between the SW and CS cohort (Table 2).

**Table 2:** Difference-in-difference generalized linear model estimates for total healthcare costs with Puhani recycled predictions (Unadjusted Model)

	QT Cohort vs. CS Cohort			SW Cohort vs. CS Cohort			
	Estimate	SE	p-value		Estimate	SE	p-value
Cohort (reference = CS)	0.48	0.02	< 0.001		0.35	0.10	< 0.001
Time (reference = pre-index period)	-0.05	0.02	0.01		-0.05	0.02	0.01
Cohort X Time	-0.04	0.03	0.16		-0.01	0.21	0.95
	**Estimated differences in changes of costs associated with changes in smoking behavior**						
Cohort (reference = CS)	-$1,532 (-$3,671, $608)	1,092	0.16		-$452 (-$15,415, $14,511)	7,634	0.95

In the Adjusted Model, COPD exacerbations and DCI were included as covariates to the DiD model to account for smoking cessation after disease diagnosis [24]. After adjusting for DCI and two COPD exacerbations, there was a statistically significant reduction in total healthcare costs of -$2,910 (95% CI: -$4,485, $-1,335) in the year following cessation for patients in the QT cohort and a similar but non-statistically significant decrease of -$5,312 (95%CI: -$11,067, $442) in the year following switching for the SW cohort (Table 3).

**Table 3:** Difference-in-difference generalized linear model estimates for total healthcare costs with Puhani recycled predictions, adjusted for COPD exacerbation and DCI (Adjusted Model)

	QT Cohort vs. CS Cohort			SW Cohort vs. CS Cohort				
	Estimate	SE	p-value		Estimate	SE	p-value	
Cohort (reference = CS)	0.35	0.02	< 0.001		0.32	0.10	< 0.001	
Time (reference = pre-index period)	-0.07	0.02	< 0.001		-0.07	0.02	< 0.001	
Cohort X Time	-0.10	0.03	< 0.001		-0.19	0.11	0.08	
Deyo-Charlson Comorbidity Index	0.24	0.003	< 0.001		0.27	0.005	< 0.001	
COPD Exacerbation	0.15	0.01	< 0.001		0.16	0.01	< 0.001	
Cohort (reference = CS)	**Estimated differences in changes of costs associated with changes in smoking behavior**							
	-$2,910 (-$4,485, $-1,335)	804	< 0.001		-$5,312 (-$11,067, $442)	2,936	0.08	

**Fig. 5:**
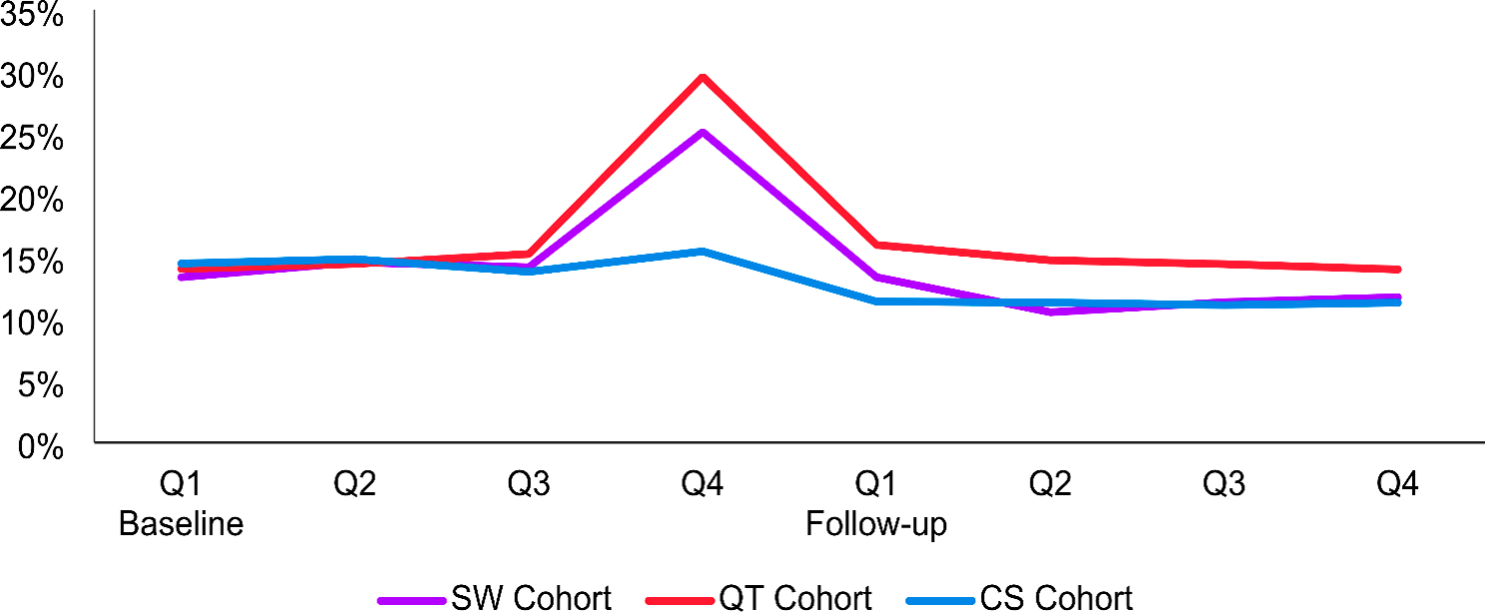
Proportion of patients with COPD exacerbations during the baseline and follow-up periods by cohort on a quarterly basis (Q)

As shown in Table 1, patients in the QT and SW cohorts had a higher prevalence of many baseline clinical characteristics including COPD exacerbation compared to the CS cohort. Inspection of COPD exacerbations on a quarterly basis showed higher prevalence of one or more COPD exacerbations in the quarter immediately before smoking behavior change among the QT and SW cohorts in the study compared to the CS cohort (Fig. 5).

Therefore, in sensitivity analysis we re-ran the unadjusted DiD models excluding 5,340 patients who had a COPD exacerbation in the 3 months prior to the index date (Sensitivity Analysis). Despite the numerically higher reductions for the SW cohort, the overall statistical inference from this sensitivity analysis (Table 4) was similar to those from the Unadjusted Model (Table 2). Due to the further reduction in the number of patients in the SW cohort (185 vs. 247 in the Unadjusted Model), the adjusted models were not replicated in Sensitivity Analysis.

**Table 4:** Difference-in-differences generalized linear model estimates for total healthcare costs with Puhani Recycled Predictions, excluding patients with COPD exacerbations 3 months prior to Index (Sensitivity Analysis)

	QT Cohort vs. CS Cohort			SW Cohort vs. CS Cohort		
	Estimate	SE	p-value	Estimate	SE	p-value
Cohort (reference = CS)	0.49	0.03	< 0.001	0.48	0.11	< 0.001
Time (reference = pre-index period)	-0.07	0.02	0.001	-0.07	0.02	0.001
Cohort X Time	-0.05	0.03	0.09	0.05	0.25	0.83
	**Estimated differences in changes of costs associated with changes in smoking behavior**					
Cohort (reference = CS)	-$2,070 (-$4,481, $340)	1230	0.09	$2,065 (-$17,606, $21,737)	10,036	0.83

## Discussion

To our knowledge this is the first study that identified individuals who switched from smoking cigarettes to using ST (moist smokeless tobacco, dip, chewing tobacco, or snus) or quit tobacco from US healthcare administrative claims data to assess the potential impact of smoking behavior change on short-term healthcare costs. Findings from this study demonstrate that, while significant challenges exist as discussed below in detail, it is feasible to use individual-level administrative claims data to assess the real-world impact of transitioning from cigarette smoking to lower risk NCP.

Among male COPD patients who smoke cigarettes, greater reductions in healthcare costs were seen in the 12 months after quitting all tobacco products compared to patients continuing cigarette smoking after assuming a modest burden of two comorbidities and exacerbations. These results are consistent with the literature that smoking cessation reduces healthcare costs among patients with COPD [27, 28]. A similar trend of greater healthcare cost reduction was observed among those who quit smoking and switched to ST in the SW cohort, but the difference was marginally statistically significant (*p* = 0.08), possibly due to the small number of patients in the ST cohort and reduction in statistical power after the introduction of covariates.

A strength of this study is the use of the DiD model where the individual serves as their own control, which accounts for time-invariant characteristics, both observed and unobserved. In addition, we adjusted for arguably two of the most relevant variables, namely, comorbidity and COPD exacerbation, to account for potential differences in health conditions across the cohorts. It is not surprising that the total number of comorbidities and COPD exacerbations were associated with an increase in direct healthcare costs. Further, after accounting for the total number of comorbidities and COPD exacerbations, the estimated total costs for COPD patients were attenuated rather dramatically. However, the estimated reduction in direct healthcare costs became more robust for COPD patients who changed their combustible smoking behavior compared to those who continued to smoke. This result cannot rule out regression to the mean for sicker COPD patients who changed their smoking behavior compared to healthier COPD patients who continued smoking as an explanation. Similar to quitting smoking, switching from cigarette smoking to ST can relieve COPD symptoms, which leads to reduction in healthcare costs among COPD patients, as ST use is associated with lower risks compared to cigarette smoking [29, 30]. Despite the plausibility underlying this observation, these results should not be interpreted as definitive evidence for causal relationships because of the observational nature of the study, the relatively small number of patients in the SW cohort, and the limitations discussed below. Studies with more robust patient numbers and appropriate longitudinal study design will be needed to further explore the impact of switching to ST on direct healthcare costs.

While a study assessing the impact of switching to a range of NCP would have more meaningful public health implications, this study focused on ST only because healthcare claim codes did not exist for modern NCP like ENDS and nicotine pouches. Some limitations of this study are inherent in any retrospective analysis using healthcare claims data. First, this study was limited to only those individuals with commercial health coverage or private Medicare coverage. Consequently, results of this analysis may not be generalizable to individuals with other insurance or without health insurance coverage. Second, the potential for misclassification of smoking and ST use status, covariates, or study outcomes was present as patients were identified through administrative claims data as opposed to medical records. As with any claims databases, the MarketScan^®^ Databases rely on administrative claims data for clinical detail. These data are subject to data coding limitations and data entry error. Observable smoking behavior change is limited to the duration of a patient’s follow-up period and is reliant upon diligent coding by the healthcare provider. In addition, due to incomplete capture of tobacco use information, it is safe to assume that some patients without any evidence of tobacco use in their claims data actually used tobacco products, which likely attenuated the effect size observed. Third, not accounting for smoking duration and intensity, which can significantly impact both the incidence and exacerbation of COPD, is another limitation of this study because such information is not well documented in claims data. Fourth, important covariates that are strongly associated with tobacco product use behaviors and smoking-related diseases (e.g., lifestyle factors, BMI, blood pressure, high cholesterol, etc.) are not captured or are limited in the claims database and not accounted for in the study. Systematic differences between patients who continue to smoke and those who quit or transitioned to ST may contribute to the differences in healthcare costs among the three cohorts (see additional discussions below). Fifth, despite the large size of the Marketscan^®^ Databases, the SW cohort is relatively small, which limited the statistical power of the analysis and the interpretation of the study findings. Finally, we only had two time points for each individual which, in combination of the variables available in the claim data, limited our ability to incorporate potential time-varying confounders in this analysis. Therefore, we could not assess whether the parallel trends assumption of DiD models was met or not. We accounted for potential factors that could lead to the violation of the parallel trend assumption in the Adjusted Model and conducted an additional sensitivity analysis given that COPD exacerbations were associated with the smoking behaviors of interest (i.e. quitting and switching). Future studies with a larger number of time points and time-varying variables will offer greater insights about the relationship between switching and healthcare costs. Nonetheless, we provided a “proof of concept” in this study and laid a foundation for future studies.

The paucity of NCP use history documentation in healthcare claims records is a major challenge for real-world evidence studies assessing the impact of transitioning away from cigarette smoking. Among tobacco use behaviors documented in claims records, the overwhelming majority are related to cigarette smoking, primarily through ICD diagnosis codes, which have been reported to have perfect specificity in identifying individuals who smoke cigarettes [31]. However, since ICD codes are used for the diagnoses of diseases rather than document tobacco use behavior per se, they have relatively low sensitivity (0.32) in identifying individuals who smoke cigarettes [31]. Combing natural language processing (NLP) of unstructured clinical notes from electronic health records (EHR) with ICD codes has been reported to substantially increase the sensitivity (0.82) of identifying individuals who smoke cigarettes using EHR [31]. Therefore, adding EHR as an additional data source and using ICD codes in combination with NLP extraction of smoking status from unstructured clinical notes will substantially enhance the identification of adults who currently smoke cigarettes as well as adults who previously smoked and quit smoking than using claims data alone. NLP extraction of information on smoking duration and intensity from clinical notes in EHR may further enhance the design of future studies. Not having standardized codes for NCP other than ST precluded the investigation of modern NCP. Nonetheless, considerable increases in the documentation of ENDS use in unstructured clinical notes over the last 10 years has been reported [32–35], with some health systems adding specific fields for capturing ENDS use in their EHR [36, 37]. While existing evidence suggests ENDS use is still substantially under-documented in EHR [33, 35, 38, 39], NLP extraction of modern NCP use data from clinical notes in EHR will facilitate assessment on the impact of switching to modern NCP. Creating standardized ICD codes for modern NCP like ENDS and nicotine pouches, broader implementation of SNOMED CT (Systematized Nomenclature of Medicine - Clinical Terms, which already include ENDS-related codes) in EHR systems, in combination with more diligent documentation of tobacco product use in medical records will greatly facilitate the assessment of the public health impact associated with modern NCP.

Dual- and poly-product use is common among adults who smoke cigarettes during their transitioning journey to NCP. For example, two studies among patients with ENDS use documentation in different EHR databases both reported that over half of the patients smoked cigarettes concurrently [34, 35]. A hybrid study design linking detailed tobacco use history information including duration, intensity, and dual/poly product use collected directly from consumers through questionnaires to existing records in EHR and/or healthcare claims represents a good option to further enhance the design of future real-world evidence studies on NCP.

Another major challenge for real-world evidence studies on transition from cigarette smoking the NCP is mitigating confounding by baseline health-related attributes that are differentially associated with various tobacco use behavior changes. Studies on patients with COPD have counterintuitively reported better outcomes for adults who currently smoke cigarettes including lower odds of COPD exacerbation than those who quit smoking [40, 41]. Higher healthcare utilization and cost around the time of smoking cessation has also been reported in the literature [25, 26]. Previous research has shown that individuals who quit smoking recently are more likely to utilize more healthcare services within a year than those who currently smoke cigarettes [23]. These results have been interpreted as evidence that recent diagnoses of major diseases often leads to cigarette cessation, which would likely attenuate the beneficial effects of quitting smoking among individuals who quit recently when assessed cross-sectionally [24] and confound studies assessing the impact of smoking behavior change for patients with symptomatic COPD [42]. At baseline, patients in the QT and SW cohorts in our study also had higher healthcare utilization rates, higher overall healthcare costs, and higher DCI scores than the CS cohort (Table 2), indicating higher comorbidities. In addition, higher proportions of patients in the QT and SW cohorts had at least one COPD exacerbation in the quarter immediately before the index dates than the CS cohort (Fig. 5). Higher numbers of baseline comorbidities and exacerbations have been found to be associated with increases in baseline direct healthcare costs [21], which is consistent with data for patients in the QT and SW cohorts in our study. Combined, these observations strongly suggest that some patients in the QT and SW cohorts in this study changed their smoking behavior after COPD exacerbation or new disease diagnosis [21], which would bias the results of the analysis by violating the parallel trends assumption for DiD models. Therefore, it is not surprising that analyses accounting for comorbidities and COPD exacerbations resulted in more robust estimated reductions in direct healthcare costs in the QT and SW cohorts compared to the CS cohort. In this study, while we showed that DiD models may be useful to control for time-invariant variables, careful consideration should be given to potential time-varying confounders in future studies.

In line with the recent FDA guidance on real-world evidence [43], we identified six areas to enhance data relevance and reliability as well as methodologies for collecting and analyzing real-world data that would allow for more robust assessments of the real-world clinical and healthcare cost impact of modern NCP that have been determined to be “appropriate for the protection of the public health” [44] and authorized to be marketed in the United States by the FDA: [1] more diligent screening and documentation of comprehensive tobacco use history information including duration, intensity, and dual/multiple product use by healthcare providers in medical records; [2] creation and adoption of new standardized codes for documenting use of modern NCP like ENDS, nicotine pouches and heated tobacco products; [3] leveraging artificial intelligence and/or natural language processing to maximize the utilization of unstructured data in EHR; [4] combining data from both EHR and claims databases to enable adjustment for important demographic attributes associated with cigarette smoking; [5] using a hybrid study design by collecting comprehensive tobacco use history directly from consumers to overcome the inherent limitations of medical records due to practitioners’ resource constraints, and subsequently linking that information to medical records; and [6] leveraging longitudinal data to better control for smoking cessation after disease diagnosis which would likely attenuate the observed beneficial effects among those who quit smoking or switched to tobacco products at the lower end of the risk continuum.

## Conclusion

Epidemiological evidence supporting a reduction in the harm caused by cigarette smoking after adults who smoke cigarettes switch to modern NCP is limited, and real-world evidence has the potential to mitigate the evidence gap. In this exploratory study with a novel design using individual-level healthcare claims data to assess the impact of transitioning away from cigarette smoking, we found evidence supporting greater reductions in direct healthcare costs among adults who smoke cigarettes with COPD after quitting tobacco or switching to ST compared to continuing smoking. While these results need to be interpreted with caution due to the limitations discussed above, and even more so for the SW cohort with a small number of patients, this study demonstrates the feasibility of using real-world data from medical records to conduct real-world evidence studies on the harm-reduction potential of NCP.

Based on the limitations, challenges and learnings identified during the study, we proposed six concrete recommendations to improve future tobacco harm reduction real-world evidence studies by integrating additional real-world healthcare data from multiple data sources.

## Electronic Supplementary Material

Below is the link to the electronic supplementary material.

Supplementary Material 1

## Data Availability

The data that support the findings of this study are available from Merative. Restrictions apply to the availability of these data, which were used under license for this study.

## Abbreviations

COPD: Chronic obstructive pulmonary disease
CPT: Current Procedural Terminology
CS: Continued smoking cohort
DCI: Deyo-Charlson Comorbidity Index
DiD: Difference in differences
EHR: Electronic health records
ENDS: Electronic nicotine delivery systems
FDA: US Food and Drug Administration
HCPCS: Healthcare Common Procedure Coding System
ICD-9/10-CM: International Classification of Diseases, Ninth/Tenth Edition, Clinical Modification; IP: inpatient
NLP: Natural language processing
QT: Quit tobacco cohort
OP: Outpatient
NCP: Non-combustible products
ST: Smokeless tobacco
SW: Switched to ST cohort
