# Supplementary material for: Assessing healthcare cost changes associated with transitioning away from cigarette smoking using healthcare claims data: an exploratory study among adult male patients with COPD
Source: Harm Reduct J. 2024 Dec 23;21:227. doi: 10.1186/s12954-024-01141-4 (PMC11665026; doi:10.1186/s12954-024-01141-4)
Supplement: Supplementary file 1 — Supplementary Material 1 [file 12954_2024_1141_MOESM1_ESM.docx]

# Supplemental File I: Description of Merative^TM^ MarketScan^®^ Research Databases

The Merative^TM^ MarketScan^®^ Commercial Database contains the inpatient, outpatient, and outpatient prescription drug experience of employees and their dependents, covered under a variety of fee-for-service and managed care health plans, including exclusive provider organizations, PPOs, POS plans, indemnity plans, and health maintenance organizations (HMOs).

Both the Merative^TM^ MarketScan^®^ Commercial and Medicare Databases provide detailed cost, use, and outcomes data for healthcare services performed in both inpatient and outpatient settings. The medical claims are linked to outpatient prescription drug claims and person-level enrollment data through the use of unique enrollee identifiers.

These databases are curated from a collection of administrative claims from large, self-insured organizations who use an Merative^TM^ decision support tool to help them manage and evaluate the experience of their enrollees. As the fiduciarily responsible party, these data contributors own these administrative claims and through the relationship with Merative^TM^ permit the aggregation and de-identification of their data to create the Merative^TM^ MarketScan^®^ Research Databases.

All database records are de-identified and fully compliant with United States patient confidentiality requirements, including the Health Insurance Portability and Accountability Act (HIPAA) of 1996. The databases have been evaluated and certified by an independent third party to follow the HIPAA statistical deidentification standard. The databases were certified to satisfy the conditions set forth in Sections 164.514 (a)-(b)1ii of the HIPAA privacy rule regarding the determination and documentation of statistically de-identified data. Because the study described herein uses only de-identified patient records and does not involve the collection, use, or transmittal of individually identifiable data, Institutional Review Board (IRB) approval to conduct this study is not necessary.

# Supplemental File II: Codes used to identify tobacco product use status

| **Code Type** | **Code** | **Description** | **Cohort** |
| --- | --- | --- | --- |
| ICD-10-CM | F17210 | Nicotine dependence, cigarettes, uncomplicated | CS |
| ICD-10-CM | F17213 | Nicotine dependence, cigarettes, with withdrawal | CS |
| ICD-10-CM | F17218 | Nicotine dependence, cigarettes, with other nicotine-induced disorders | CS |
| ICD-10-CM | F17219 | Nicotine dependence, cigarettes, w unspecified nicotine-induced disorder | CS |
| ICD-10-CM | F17220 | Nicotine dependence, chewing tobacco, uncomplicated | SW |
| ICD-10-CM | F17223 | Nicotine dependence, chewing tobacco, with withdrawal | SW |
| ICD-10-CM | F17228 | Nicotine dependence, chewing tobacco, with other nicotine-induced disorders | SW |
| ICD-10-CM | F17229 | Nicotine dependence, chewing tobacco, with unspecified nicotine-induced disorder | SW |
| ICD-10-CM | Z87891 | Personal history of nicotine dependence | QT |
| ICD-9-CM | 3051 | Tobacco use disorder | CS |
| ICD-9CM | V1582 | Personal history of tobacco use, presenting hazards to health | QT |
| CPT | 1034F | Current tobacco smoker | CS |
| CPT | 1035F | Current smokeless tobacco user | SW |
| HCPCS | G8455 | Current tobacco smoker | CS |
| HCPCS | G9902 | Patient screened for tobacco use and identified as a tobacco user | CS |
| HCPCS | G8456 | Current smokeless tobacco user | SW |
| HCPCS | G8688 | Currently a smokeless tobacco user (eg, chew, snuff) and no exposure to secondhand smoke | SW |
| HCPCS | G8692 | Current smokeless tobacco user (eg, chew, snuff) and no exposure to secondhand smoke | SW |

Supplemental File III: All-cause healthcare utilization in baseline and during follow-up

All-cause healthcare utilization included inpatient admissions, outpatient services (i.e., emergency room, outpatient office, laboratory, radiology, and other outpatient), and outpatient pharmacy prescriptions. A patient was considered to have type specific healthcare utilization if any (i.e., ≥1) inpatient admission, emergency room visit, outpatient service, laboratory service, radiology service, or outpatient prescription was identified.

## *Healthcare utilization*

Consistent with higher prevalence of certain conditions and DCI scores shown above, baseline healthcare utilization in both SW and QT cohorts was higher than in the CS cohort, particularly for inpatient and emergency services (Table S1). 42.3% of the QT cohort and 37.7% of the SW cohort had at least one inpatient admission in the baseline period, compared to 24.2% for the CS cohort. Similarly, 44.2% and 48.6% patients in the QT and SW cohorts, respectively, had at least one emergency room visit during baseline, compared to 35.0% for the CS cohort. Over 90% of patients in all cohorts had an outpatient office visit in the baseline period, as well as over 97% of patients having at least one outpatient pharmacy claim.

In the follow-up period, while healthcare utilization remains numerically higher among the QT and SW cohorts compared to the CS cohort for all service categories, the differences narrowed for inpatient admission and emergency room visit compared to baseline. Healthcare utilization was not meaningfully different between patients in the SW and QT cohort in both the baseline and follow-up periods.

Table S1. Healthcare utilization in the baseline and follow-up period, by cohort

|  | SW Cohort | QT Cohort | CS Cohort |
| --- | --- | --- | --- |
|  | N = 247 | N = 12,013 | N = 11,167 |
|  | N (%) | N (%) | N (%) |
| Baseline Utilization |  |  |  |
| Inpatient | 93 (37.7%) | 5,078 (42.3%) | 2,706 (24.2%) |
| Emergency Room | 120 (48.6%) | 5,306 (44.2%) | 3,913 (35.0%) |
| Outpatient Office | 234 (94.7%) | 11,431 (95.2%) | 10,259 (91.9%) |
| Laboratory | 212 (85.8%) | 10,522 (87.6%) | 9,243 (82.8%) |
| Radiology | 204 (82.6%) | 10,860 (90.4%) | 8,382 (75.1%) |
| Other Outpatient Service | 238 (96.4%) | 11,676 (97.2%) | 10,354 (92.7%) |
| Outpatient Pharmacy | 245 (99.2%) | 11,756 (97.9%) | 10,869 (97.3%) |
| Follow-up Utilization |  |  |  |
| Inpatient | 55 (22.3%) | 2,978 (24.8%) | 2,117 (19.0%) |
| Emergency Room | 83 (33.6%) | 3,985 (33.2%) | 3,464 (31.0%) |
| Outpatient Office | 230 (93.1%) | 11,228 (93.5%) | 9,868 (88.4%) |
| Laboratory | 207 (83.8%) | 10,163 (84.6%) | 8,823 (79.0%) |
| Radiology | 192 (77.7%) | 9,775 (81.4%) | 7,788 (69.7%) |
| Other Outpatient Service | 228 (92.3%) | 11,366 (94.6%) | 9,986 (89.4%) |
| Outpatient Pharmacy | 239 (96.8%) | 11,743 (97.8%) | 10,726 (96.1%) |
